# A new physical mapping approach refines the sex-determining gene positions on the *Silene latifolia* Y-chromosome

**DOI:** 10.1038/srep18917

**Published:** 2016-01-08

**Authors:** Yusuke Kazama, Kotaro Ishii, Wataru Aonuma, Tokihiro Ikeda, Hiroki Kawamoto, Ayako Koizumi, Dmitry A. Filatov, Margarita Chibalina, Roberta Bergero, Deborah Charlesworth, Tomoko Abe, Shigeyuki Kawano

**Affiliations:** 1 RIKEN Nishina Center, 2-1 Hirosawa, Wako, Saitama 351-0198, Japan; 2 Department of Integrated Sciences, Graduate School of Frontier Sciences, The University of Tokyo, Kashiwa, Chiba 277-8562, Japan; 3 Department of Plant Sciences, University of Oxford, South Parks Road, Oxford OX1 3RB, UK; 4 Institute of Evolutionary Biology, University of Edinburgh, School of Biological Sciences, Edinburgh EH9 3JT, UK

## Abstract

Sex chromosomes are particularly interesting regions of the genome for both molecular genetics and evolutionary studies; yet, for most species, we lack basic information, such as the gene order along the chromosome. Because they lack recombination, Y-linked genes cannot be mapped genetically, leaving physical mapping as the only option for establishing the extent of synteny and homology with the X chromosome. Here, we developed a novel and general method for deletion mapping of non-recombining regions by solving “the travelling salesman problem”, and evaluate its accuracy using simulated datasets. Unlike the existing radiation hybrid approach, this method allows us to combine deletion mutants from different experiments and sources. We applied our method to a set of newly generated deletion mutants in the dioecious plant *Silene latifolia* and refined the locations of the sex-determining loci on its Y chromosome map.

Chromosomes of animals, plants, and fungi generally include non-recombining regions surrounding the centromeres, but non-recombining regions occur in other genomic regions, including sex-linked regions. Compared with recombining regions, there are major difficulties for assembly of genome sequences, as they generally accumulate repetitive sequences, which renders the determination of gene order or the assembly of genomic scaffolds problematic, and genetic mapping to aid assembly is not possible. Physical mapping using deletions is a helpful approach, and several approaches have been developed, but these suffer from several drawbacks. Radiation hybrid (RH) mapping has been very useful in mammals, such as humans^1^ and dogs^2^, and in other animals (e.g. the sea bream^3^). It is based on the frequencies of double-strand breaks between genetic or cytological markers after irradiation with a specific radiation dose^4^, and the dose dependency prevents the use of deletion mutants from different experiments. Methods that are independent of a specific dose and type of irradiation (or mutagen) that induces breaks are also required. Indeed, it would be desirable to use different methods, which can increase confidence in the accuracy of the maps. Another major problem is that interpretation of deletion mapping data is often not straightforward, particularly when multiple deletions may arise in a single deletion strain or individual. Data are generally analysed by attempting to minimise the total number of chromosome breaks and number of deletions assigned to each mutant, but this is usually done manually and without a principle guaranteeing that the best solution has been found.

Here, we present a new mapping software (DelMapper) for interpreting deletion data using a novel scoring system to obtain the best order of the markers using an approach that employed the traveling salesman problem (TSP), which is often used in radiation hybrid mapping^5^,^6^. We evaluated the effects of the numbers of mutants, markers, and deletions on the accuracy of DelMapper. Our approach allows deletion mutants from different experiments and sources to be combined, and is suitable for physical mapping in any organism.

We applied our approach to refine the map of the Y chromosome of a dioecious plant, *Silene latifolia*, which has heteromorphic sex chromosomes (X and Y). Most of the Y chromosome is non-recombining^7^,^8^,^9^ and DNA sequence divergence between X- and Y-linked sequences indicates that recombination suppression evolved in at least two events, the first of which occurred between 5 and 10 million years ago^10^,^11^. Thus, this sex chromosome system is much younger than the well-studied systems in mammals, birds, or fruit flies, and is well suited for studying the early stages of sex chromosome evolution. The non-recombining region includes at least two sex-determining loci, a gynoecium suppression function (GSF) and a stamen promoting function (SPF). These loci were initially inferred by crosses between dioecious plants and related species, and by studying deletions of parts of the Y chromosome found in asexual and hermaphroditic mutants (deleted for SPF and GSF factors, respectively, as reviewed in ref 9); more recent work, with more flower phenotype mutations, confirmed these conclusions^12^,^13^.

The *S. latifolia* Y chromosome is gene-rich compared with those of mammals or fruit flies, and many Y-linked genes identified by cloning appear to be intact, complete copies with X-linked counterparts, suggesting that genetic degeneration of this Y is much less extensive than that of mammalian Ys^10^,^14^,^15^,^16^,^17^,^18^,^19^,^20^,^21^. Using recent advances in next-generation sequencing, new studies have identified several hundred Y-linked genes^22^,^23^,^24^. However, such studies also demonstrate that the Y has lost genes^25^ and it is estimated that the coding sequences of as many as 23% of the remaining Y-linked genes include premature stop codons^26^. However, rearrangements with respect to the X cannot currently be analysed, and it is unknown whether Y degeneration involved deletions, perhaps of large regions including multiple genes. If chromosomal inversions were involved in the recombination suppression, the inverted regions should correspond to distinct regions with different levels of DNA sequence divergence, as in papaya^27^. In the absence of a physical map, this cannot be tested in *S. latifolia*.

Deletion mapping can be used to define the locations of Y-linked sex determining loci. Lebel-Hardenack *et al.*^28^ conducted the first experiment to map the *S. latifolia* Y chromosome using molecular markers (mostly AFLPs) in combination with deletions induced by ionising radiation. Their map was inferred using the RHMINBRK software included in the RHMAP package for RH mapping^29^. The map was constructed using the minimum number of obligate chromosome breaks criterion by the simulated annealing strategy^30^,^31^ and was refined by Moore *et al.*^21^. Zluvova *et al.*^32^ also mapped the Y chromosome by minimising the number of chromosome breaks. These maps were consistent with those from several other studies^33^,^34^,^35^,^36^. However, simulated annealing, despite being based on a maximum likelihood approach, does not guarantee that the best solution is found^29^. Moreover, these approaches encounter problems as the number of markers increases, and maximising the likelihood requires substantially more time than minimising the number of chromosome breaks^29^. For high-throughput data, which should become available soon, computational efficiency is critical. Our new approach combines the criterion of the minimum number of obligate chromosome breaks with the branch-and-bound strategy, which guarantees that the best solution is found^37^ and improves the efficiency of the calculation by clustering markers. We verified the approach using simulations, which also provide an approach to estimating the accuracy expected for a given data set. Our approach allows us to integrate the complete physical map of the *S. latifolia* Y chromosome and to use multiple genic markers to locate the sex-determining regions, SPF and GSF, more accurately than was previously possible.

## Results

### Screening mutants used for our new mapping

**Figure 1:**
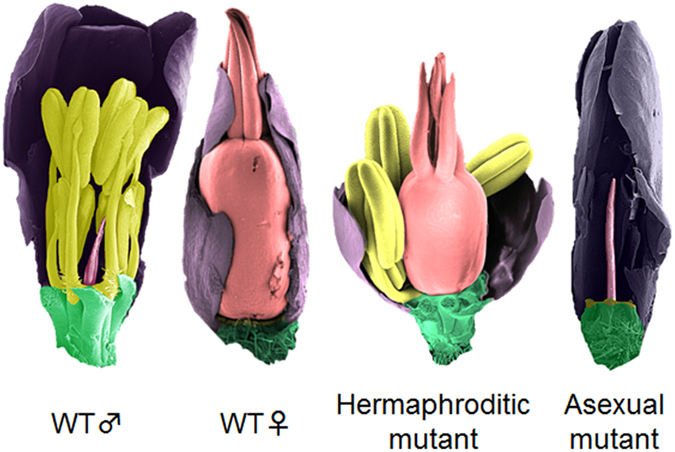
Scanning electron micrographs of *S. latifolia* flowers. Gynoecia, stamens, petals, and receptacles are coloured pink, yellow, purple, and green, respectively. Hermaphroditic and asexual mutants are considered to lack GSF and SPF, respectively.

To generate a set of deletion mutants for physical mapping, we irradiated seeds and pollen of an inbred *S. latifolia* line^38^ with carbon ion beams with linear energy transfer (LET) of 30 keV μm^−1^ or γ-rays, and grew 2722 plants of the M1 generation, which were screened to find hermaphroditic and asexual mutants, which are expected to have deletions in the Y-linked GSF and SPF, respectively (Fig. 1). One aim of this study was to map both sex-determining regions on the Y chromosome. Details of the mutant screening are provided in the Supplementary Methods and Supplementary Table 1. In total, we generated 41 mutants, including 15 hermaphrodites, one female-like mutant, 10 asexuals, 14 mutants with non-maturing anthers, and one male plant. These mutants were genotyped for the presence/absence of deletions at 71 Y-linked markers, as described in the Supplementary Methods. This data set was used for our new Y deletion mapping (see below).

### Tests of our new mapping method on computer-generated data sets

Building a physical map requires finding the “best” ordering of the deletions along the chromosome. This can be achieved by minimising the number of chromosome breaks in the set of deletion mutants, which reduces the ordering of deletions to the traveling salesman problem (TSP, see Methods). We created a software package, DelMapper, to solve this problem. Briefly, in a data set in which all mutants were tested, their presence or absence on all markers is treated as a mutant-by-marker matrix (Supplementary Fig. 1). Instead of assessing the number of chromosome breaks (*total cost*) in all permutations of the markers, the computational effort is reduced by clustering the markers into *n* clusters (where *n* can be specified by the user). After finding the best order of the clusters (with lowest *total cost*), DelMapper orders the markers within each cluster. To evaluate the accuracy of our approach, we created a Perl script, DelMapMaker (see Methods) for simulations and first simulated deletion data with 71 markers, 41 mutants, and 442 deleted elements, the same numbers as in our data set to be analysed (which is described in the section *Deletion map of the S. latifolia Y chromosome*). We first confirmed that all 20 simulated deletion data sets successfully reconstructed by DelMapper with our clustering procedure (number of clusters: *n* = 12) yielded the correct marker orders. Ideally, a single best map is found; however, multiple maps can have the same score as the correct map. Therefore, in the analyses below, we report counts of the number of candidate best maps.

**Figure 2:**
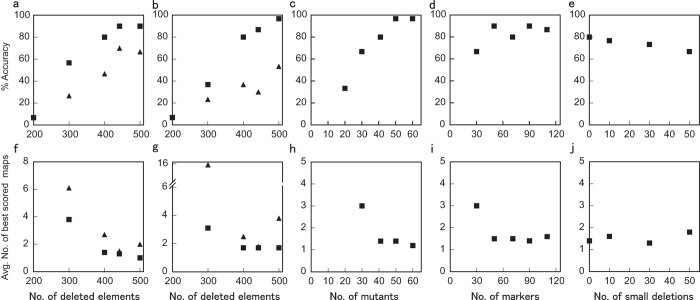
Accuracy of mapping using DelMapper. Simulated virtual maps were used as input for the DelMapper program. (**a**–**e**) Scatter plots for the accuracy (defined as the percentages of trials that successfully reconstructed the input marker order; see Methods) with a different number of deleted elements in the non-biased dataset (**a**) and biased dataset (**b**), with a different number of mutants genotyped (**c**), with a different number of markers (**d**), and with a different number of additional small deletions (**e**). (**f**–**j**), Scatter plots for the average numbers of maps having the best score with a different number of deleted elements in the non-biased dataset (**f**) and biased dataset (**g**), with a different number of mutants genotyped (**h**), with a different number of markers (**i**), and with a different number of additional small deletions (**j**). Squares and triangles indicate results using the “Any” option and “Del” option, respectively.

We first examined the effect of different total numbers of deleted elements (200, 300, 400, 442, or 500) in the data matrix, assuming that deletions occur randomly and are not biased towards specific regions or markers. As expected, the accuracy of our estimates increased, and the average number of maps with the best score decreased, with increasing numbers of deleted elements (Fig. 2a, f). When 400 deleted elements were allowed, the accuracy was 80% (Fig. 2a).

An important aspect of the scoring concerns the status of the ends of the region being mapped. We tested two alternative options for the ends of the region (see Supplementary Fig. 1). The “Del” option allows markers that are deleted in all the phenotypic mutants to be assigned to locations at the ends of the mapped region. However, this ignores the possibility that there could be a bias towards deletions of certain markers, as with maps constructed from collections of mutants with specific phenotype changes from wild-type (for example in our datasets in Supplementary Fig. 2a). In such cases, when a subset of markers is deleted in most or all the mutants, the “Del” option tends to incorrectly assign the deleted elements to the “0 end” of the map. We therefore also included an alternative option (“Any”) that is designed to be tolerant to biased mutant sets. When the two options were tested, the “Any” option proved to give higher accuracy in non-biased deletion data sets (Fig. 2a; dataset examples in Supplementary Fig. 2b. The lower accuracy under the “Del” option may be due to chance occurrence of maps with biased deletion sets in our simulations, as it was most obvious in the biased deletion mutants set, see Fig. 2b). The average number of maps with the best score using the “Any” option also tended to be smaller than that with the “Del” option (Fig. 2g). The following tests therefore used the “Any” option.

The number of mutants studied also affects the accuracy of DelMapper, as does the number of markers. For the simulations testing these effects, we used data matrices with the proportion of deleted elements (out of all the elements in each matrix) set to 0.1374 (matching the data set in Fig. 2a that has 400 deleted elements). Accuracy increased, and the number of different maps with the best score decreased, as the average number of mutants analysed was increased from 20 to 60 (Fig. 2c,h). With this proportion of deleted elements, and with 41 mutants analysed, the number of markers had less effect on the map; the accuracy and average number of maps with the best score were similar to those with all 71 markers, unless we reduced the marker number to as low as 30 (Fig. 2d,i).

Finally, we explored whether DelMapper is robust to noise, such as the presence of small deletions that do not contribute to mapping, or PCR errors. We simulated data with 400 deleted elements, 41 mutants, and 71 markers, as before, but changed the matrices by adding small deletions as PCR errors. The accuracy and the average number of the maps with the best score were only slightly affected (Fig. 2e,j).

### Deletion map of the *S. latifolia* Y chromosome

To compare the performance of our approach with previous mapping, we used the data from the *S. latifolia* Y chromosome published by Zluvova *et al.*^32^ to reconstruct the physical map. With the “Del” option (see above), DelMapper gave the same map as that of Zluvova *et al.*^32^ (Supplementary Fig. 3), with five other maps having an equally good score. This is not surprising, since the accuracy of this dataset, based on our simulations, is expected to be ~77%.

**Figure 3:**
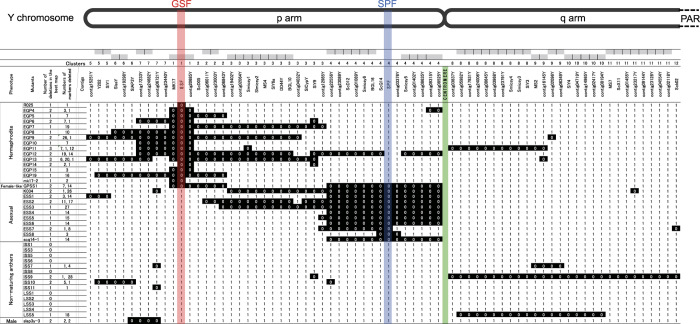
Deletion map of the constructed Y chromosome with our new mapping algorithm using either the “Del” or “Any” options. The map is based on data derived from deletions induced in inbred-line K plants, and includes 10 asexual mutants, one female-like plant, 15 hermaphrodites, 14 mutants with non-maturing anthers, and one irradiated plant with a male phenotype, as indicated in the left-hand columns. A total of 71 markers (shown in the columns to the right) were genotyped by PCR analysis in these plants. The clusters of marker loci described in the text are shown in the row above the marker names. The closely linked marker sets are coloured grey. The individual marker orders in these sets were not fixed by this analysis because the markers had the same (or extremely similar) deletion status. GSF; gynoecium suppressing function, SPF; stamen promoting function, PAR; pseudo autosomal region.

We also estimated a new deletion map of the *S. latifolia* Y chromosome with 41 newly identified mutants described above, and increased numbers of marker genotypes in the DelMapper input matrix, which included a total of 442 deleted elements. Our simulations predicted an accuracy of map reconstruction for such a dataset of 90%. Both the “Del” and “Any” options in DelMapper consistently yielded the same map for the *S. latifolia* Y chromosome. In hermaphroditic and asexual mutants, the GSF state and SPF states were respectively set to “deleted”, as previously^32^,^33^, and the female-like mutant was assumed to have both GSF and SPF deleted, whereas the 14 mutants with non-maturing anthers and the one male plant were assumed to have normal Y chromosome sex-determining genes (though deletions of Y-linked molecular markers were detected in some of these plants (see Fig. 3). Of the 26 mutants with sex phenotype changes, and therefore putatively with deletions of sex-determining genes, 25 had deleted molecular markers (in addition to GSF or SPF).

**Figure 4:**
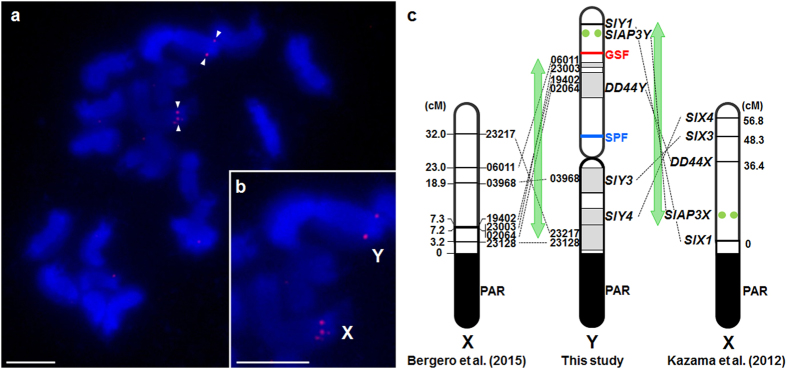
A new physical mapping approach refines the position of sex-determining genes on the *Silene latifolia* Y-chromosome. (**a**) FISH analysis of male early metaphase chromosomes using *SlAP3* probes. Male mitotic metaphase chromosomes hybridised with digoxigenin (DIG)-labelled *SlAP3* probes (red). Chromosomes were counterstained with DAPI (blue). (**b**) Magnified image of the X and Y chromosomes. Bars = 5.0 μm. (**c**) Large pericentric inversion on the Y (or X) chromosome (represented by green arrows) detected by comparing locations of genes between X and Y chromosomes. The positions of Y-linked genes of our new map were compared with their corresponding X-linked copies. The left and right X maps are derived from Bergero *et al.*^14^ and Kazama *et al.*^42^, respectively, and were obtained using different mapping populations. Their corresponding Y-copies were mapped by DelMapper in this study. FISH signals of *SlAP3X/Y* are indicated by green circles; the genetic location of *SlAP3X* is derived from Ishii *et al.*^41^ PAR; pseudo autosomal region.

Because the centromere cannot be deleted, no deletions can straddle both Y chromosome arms (the *q* arm and the *p* arm that does not include the PAR^39^). Therefore, Fig. 3 indicates that the centromere must lie between the markers in contigs 08525Y and 03837Y; below, we present evidence against the reverse marker order on the *q*-arm. The map of the putative *p* arm (the Y chromosome arm that does not include the PAR) appears to be well resolved. MK17 and ScQ14 were identified as the markers closest to the GSF and SPF genes, respectively, as in previous maps^32^,^40^. Our new map locates GSF between MK17 and genic contig20685Y in an interior region of the chromosome. The *SlAP3Y* gene maps between GSF and the inferred end of the Y*p* arm (Fig. 3). To further test the interior location of GSF and determine the centromeric end of the Y*p* arm, we located the *SlAP3Y* gene using FISH analysis with a probe cocktail specific to the *SlAP3* genome region, which can detect both X- and Y-linked alleles (see Supplementary Methods). Consistent with the Y deletion map, signals were detected near the tips of the Y and X chromosome arms Y*p* and X*q*, opposite the PAR end (Fig. 4a,b). The difference from previous FISH mapping, which concluded that it was located near the centromere^41^, is that, unlike the probe used here, the probe region used previously contained repetitive sequences, as revealed by sequencing BAC clones containing *SlAP3Y* and *SlAP3 X *42.

SPF maps between ScQ14 and the genic contig 03376Y. Thus, GSF and SPF are flanked by different markers. Our map also orders the genes and markers between the GSF and SPF regions, including 30 of our newly mapped genic markers (Fig. 3). These genic markers greatly improve our ability to locate the GSF and SPF regions, compared with previous maps. The observation that the entire region is deletable suggested that the region does not include any genes whose deletion from the Y causes dominant lethality.

Unexpectedly, 10 of the 14 mutants with non-maturing anthers had no deleted markers, a larger proportion than in previously published studies of deletions in this species^28^,^32^. The marker density around the putative gene(s) whose deletion causes the non-maturing anther phenotype may be too low to detect deletions, or the only viable deletions may be very small and contain only the gene affecting anther maturation; however, the latter possibility seems unlikely, as the deletion of a small Y-linked region should generally be recessive in its effect on viability, due to the presence of a functioning X-linked allele. The K-line is possibly heterozygous for a mutation at an autosomal gene involved in anther maturation, and our mutants have mutations in the functioning allele; however, 10 of these events seems unlikely.

### Rearrangement of the *S. latifolia* Y chromosome

Our new map includes 11 genes whose X-linked copies have been genetically mapped: seven by Bergero *et al.*^25^ and four by Kazama *et al.*^43^. The orders of these genes on the Y chromosome in Fig. 3 differ from their ordering on the X chromosome map (Fig. 4c). For example, contigs 06011Y and 23003Y, which map in the same marker cluster of the Y chromosome, are somewhat proximal to the GSF factor in Figs 3 and 4, but their X-linked copies are on the X region near the PAR, consistent with the Y chromosome pericentric inversion previously inferred^44^,^45^,^46^, which includes SPF. We conclude that this inversion is larger than previously thought and includes *SlY1*, which was moved to the distal end of the Y*p* arm by the inversion, but is close to the PAR in the X genetic map. Even if the *q*-arm marker order is the reverse of the order described above, a large pericentric inversion that includes SPF and *SlY1* is still possible; however, this order of markers requires a much more complicated scenario of rearrangements and, therefore, seems unlikely.

## Discussion

The chief advantage of the new approach for map analysis implemented in DelMapper is that it allows map construction from mutants in non-recombining genome regions regardless of the mutagens used and of the doses employed (including spontaneous mutations). Thus, mutants generated in different studies or different laboratories can be combined to make best use of the time consuming collection of mutants. Non-recombining genome regions include not only sex-linked regions, such as the one studied here, but also include pericentromeric regions, which can form large non-recombining regions in plant chromosomes, and can contain large proportions of genes in some plants^47^,^48^,^49^ (including the regions controlling the self-incompatibility genes of some plant species^50^) and centromere-linked regions in which mating type loci are located in some fungi^51^. Many interesting and important genes are located in non-recombining regions, and mapping such regions is necessary to aid assembly of *de novo* sequences of the region to identify these genes and study their evolution. Examples include some apomixis genes^52^, “supergenes”, such as the large non-recombining “social chromosome” in the fire ant^53^, the multi-gene regions controlling possible Batesian mimicry in some butterflies^54^, and the genome regions controlling the two flower morphs and self-incompatibility in distylous plants^55^,^56^.

DelMapper allows map construction to be tailored to cases when the region to be mapped is flanked at both ends by recombining genome (such as pseudoautosomal regions at both ends of a sex chromosome pair, as in spinach^57^, papaya^58^, or asparagus^59^), so that neither end can have the deleted status or if the end is known to be deleted. The approach proved reliable and accurate in simulations, and is robust to PCR errors (Fig. 2e,j). The accuracy depends strongly on the number of mutants in the dataset and the total number of deletions, but less on the number of markers used (Fig. 2).

In our map (as in those previously published), it was necessary to allow multiple deletions in individual mutants. The high repetitive sequence content of this Y chromosome may cause it to have many weak sequences that break readily. We therefore examined the data to determine whether multiple breaks tend to occur in specific locations, but found no clear evidence for this, although multiple mutants with similar deletion states were obtained in the region around the SPF gene (between marker 22556 and contig 08525Y).

Our *S. latifolia* Y deletion map confirms that a large pericentric inversion occurred on the Y chromosome during sex chromosome evolution. The *S. latifolia* X chromosome fully sex-linked region (corresponding to the mapped region in Fig. 4c) is divided into two regions with different silent site sequence divergences, called “evolutionary strata”, indicating that recombination suppression with the Y chromosome evolved in two distinct events, an older event that created stratum 1 and a later one creating stratum 2^11^. The large inversion includes genes that map to both strata 1 and 2 on the X chromosome^25^, so it probably occurred after stratum 1 formed. It is unknown how stratum 1 evolved, but further inversions must have occurred in the Y chromosome of the K-line, as previously inferred in other *S. latifolia* genotypes^44^,^46^. However, several studies suggested that the orders of Y-linked markers differ between genotypes, and Y chromosomes from different *S. latifolia* individuals thus appear to have different arrangements^33^,^45^. The Y chromosome maps of the U strain^21^,^28^,^32^,^33^ and the IL160 plant^45^ are identical, as far as can be determined, with *SlCypY*, *DD44*, and *SlssY* on the *p* arm of the Y chromosome. Our map of the inbred line “K” is similar to this map, in which *SlY3* and *SlY4* are located on the Y*q* arm between the centromere and the PAR, while *SlY1* is located near the end of the Y*p* arm. Yet another strain, IL25 from the UK, has *SlY3* and *SlY4* located on the Y*p* arm and might have another Y inversion^45^. In the M strain^21^,^28^,^32^,^33^ and the “French” strain^33^, *SlY1* is located near the PAR. The difference of mapping data between individuals should be considered in dealing with our current map.

Our Y chromosome deletion mutants were induced by either heavy-ion or gamma-ray irradiation, and the former has high LET and should induce more localised deletions than low-LET radiation (X-rays or γ-rays), based on results in *Arabidopsis thaliana*^60^,^61^. However, deletion sizes in *A. thaliana* chromosomes are restricted by the presence of essential genes at a high density, including genes necessary for pollen functioning (large deletions cannot be transmitted to the progeny generation^62^), and possibly genes where two copies are required for zygote viability. In contrast, non-recombining regions such as the *S. latifolia* Y chromosome are predicted to accumulate repetitive sequences^63^ and the Y is indeed much larger than the X chromosome^64^, and there is also direct evidence for the presence of repetitive sequences^65^,^66^,^67^. It may thus have a low density of essential genes, and there is evidence for loss of some genes^25^. Our mapping data revealed that both a 30.0-keVμm^−1^ heavy-ion beam and γ-irradiation can co-delete large numbers of markers (potentially causing loss of physically large Y chromosome regions), as well as cause small deletions. Locating deletions should help to identify regions of the Y chromosome containing essential genes that are required for viability or for male fertility. If the Y*q* arm stopped recombining more recently than the Y*p* arm, one would expect it to have smaller deletions, but we found no significant size differences (Fig. 3). Every marker was deleted in at least one mutant., which suggests that the Y chromosome includes no regions that cannot be deleted, since the markers were not in pre-selected genes known to be potentially deletable, but were chosen randomly from Y-linked sequences (see Methods). This suggests that mapping using flower phenotype mutants should be adequate to reveal the organisation of this Y chromosome.

Large deletions are very helpful for developing accurate maps. In our map, large deletions in a few mutants located the GSF and SPF factors in the Y map (Fig. 3). However, to identify the individual genes, small deletions are required. Two of our hermaphroditic mutants, EGP14 and mk17-2, have small deletions that include the GSF region and one marker on either side, and in one hermaphrodite, R025, no deletion was detected. Similarly, one asexual mutant (ESS8) has a small deletion pinpointing the SPF region. Candidate sex-determining genes for further testing can potentially be found by identifying the genes in these deleted regions, provided that the regions do not include too many genes.

## Methods

### Mutant screening and genotyping

The mutant screening, observation by scanning electron microscopy of the wild-type and mutant flowers (Fig. 1), genotyping of Y-linked markers, and FISH analysis are described in the Supplementary Methods.

### Deletion mapping using the travelling salesman problem

The travelling salesman problem (TSP) is an NP-hard problem in combinatorial optimisation^68^,^69^. Ben-Dor *et al.*^5^ described the TSP as follows: a salesman has to visit *n* cities exactly once and then return to the first city. The goal is to find the path that minimises the total cost of the tour (the sum of the costs of travelling along each edge of the path, measured in terms of the numbers of breaks involved in each path). For the case of deletion mapping, the analysis was performed as follows. In step (i), each marker was regarded as a city, and the *cost* between markers *i* and *j* was defined as the following sum:


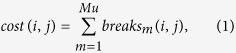


where *Mu* is the total number of deletion mutants studied, and the quantity *breaks* was set to “0” if the adjacent markers *i* and *j* were either both present or both deleted in a mutant, and to “1” if *i* was present but *j* was deleted, or *vice versa*. In step (ii), all possible permutations of the markers are considered. One end of the mapping region is assumed to be present in all mutants. In each permutation, a virtual marker representing this end of the mapping region was added before the first marker, and another virtual marker, representing the opposite end of the mapping region, after the last marker. We considered two options for the latter virtual marker: under the “Del” option, it is assumed to be deleted in all the mutants, whereas under the “Any” option, it has the same deletion status as the adjacent marker. For each permutation, step (iii) calculates the *total cost* as follows:


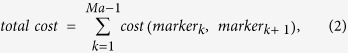


where *Ma* is the total number of markers, and *marker*_*k*_ represents the k-th marker in the permutation. Finally, step (iv) finds the permutation(s) with the lowest *total cost*.

To apply the TSP to our data, the PCR results were summarised in a matrix for input to DelMapper coded in C++ and Perl. The first row of Supplementary Figure 1a includes the marker names, and the first column the mutants’ names. The other elements indicate the deletion states (“0” when a marker is “deleted” or “1” when it is “present”). Because the number of permutations required to solve the TSP increases superpolynomially with the number of markers, we first clustered the markers into 12 clusters, each including at least one marker (Supplementary Figure 1b). A distance matrix was first computed from the input matrix by the “dist” function (method: “binary”) in the R (v3.1.0) environment. This matrix was then input as the dissimilarity structure for clustering by the “hclust” function (method: “ward.D2”) from the “stats” package in the R environment. All markers in a cluster were then initially treated as a single virtual marker (Supplementary Figure 1c). Whereas an actual marker can have only two states (0 or 1), a virtual marker representing a cluster can have multiple states, and the number of states depends on the number of present actual markers in the cluster; for example, for a cluster of three markers, the state can be “0” (all three markers deleted), “1” (one marker present), “2” (two markers present), or “3” (all markers present).

To input the clustered data into DelMapper, a new matrix was made in which the first and second rows respectively contain the names of the virtual markers (clusters) and the numbers of actual markers in each cluster (*num* values, where *num* ≥1), and the last column contains the names of mutants. The other elements indicate the states of markers. If a state is “*num* – 1” (and if *num* >3), the single deletion in the cluster is treated by the program as a PCR error, and the state is changed to “*num*”. To calculate the *cost* value of a change in state between the *k*-th marker *marker*_*k*_ and the adjacent marker, *marker*_*k+1*_, as defined above, the program sets the quantity *breaks*_*m*_*(k, k + 1)* as follows: (1) When *k* = 1, *marker*_*k*_ is classified as present (because it is the virtual marker representing the end of the mapping region). If the state of *marker*_*k+1*_ is equal to *num*_*k+1*_ (meaning that all the actual markers in this cluster are present), *breaks*_*m*_ *(k, k + 1)* is set to 0, and *marker*_*k+1*_ is classified as present in the next step; if its state is less than *num*_*k+1*_ (meaning that all or part of the actual markers in this cluster are deleted), *breaks*_*m*_*(k, k + 1)* is set to 1, and *marker*_*k+1*_ is classified as deleted in the following step (Supplementary Fig. 4a). (2) When *k* > 1, if *marker*_*k*_ is classified as present in the preceding step, both *breaks*_*m*_*(k, k + 1)* and *marker*_*k+1*_ are determined in the same manner as in step (1). If *marker*_*k*_ is determined to be deleted in the preceding step, then, if the state of *marker*_*k+1*_ is > 0, then *breaks*_*m*_*(k, k + 1)* is also set to 1, and *marker*_*k+1*_ is treated as present in the next step; if its state is 0, then *breaks*_*m*_*(k, k + 1)* is set to 0, and *marker*_*k+1*_ is treated as deleted (Supplementary Fig. 4b).

After calculating the best permutation(s), local mapping of the actual markers in each cluster is performed recursively in the same manner, except that the two virtual markers that represent the adjoining clusters of the focal cluster in the best permutation of the clusters are added to the first and last of each permutation in step (ii) (Supplementary Figure 1d). To reduce the cost of calculation, the branch and bound algorithm is used^70^.

### Computer-generated deletion “data”

Deletion data for testing our method were randomly generated using a perl script, DelMapMaker. The numbers of markers and mutants, the number of deleted elements out of all elements in the matrix, and the user-selectable option for “biased” map (to represent the situations in which mutants with a specific phenotype were tended to be collected) were used as inputs. A matrix was created in which the first row included the marker names, and the first column the names of the mutants. The other elements of the matrix were set to “1” (all markers present). For each mutant, the simulation selected the neighbouring two markers randomly if the option for “biased” map was off; if the option was on, the deleted marker positions were selected randomly, assuming a mean in the centre of the map and a standard deviation equal to half the number of markers, and the states of the corresponding elements were changed to “0” (deleted). After assigning the deletion states in each mutant, the randomly chosen numbers of elements neighbouring the deleted elements were also deleted. If the total number of deleted elements exceeded the input number, the last operation was re-done until the total number of deleted elements equalled the input number.

### Evaluating the accuracy of mapping by DelMapper using simulated data sets

To evaluate the accuracy of our approach, we simulated deletion data with different total numbers of deleted elements in the entire data set and different numbers of mutants or markers analysed. Because DelMapper tests all marker permutations, the input marker order does not affect the results. We used four sets of tester maps as follows. (1) To test the method when deletions are biased towards specific markers (because mutants with specific phenotypes were collected for study), we created “biased” and “non-biased” maps, each with 71 markers and 41 mutants and with different total numbers of deleted elements (200, 300, 400, 442, or 500) for the set of all plants ascertained as having mutations. (2) To evaluate the influence of the number of mutants on DelMapper, we created maps with 71 markers and a total of 400 deleted elements, but with different numbers of mutants analysed (20, 30, 41, 50, or 60). (3) To evaluate the influence of the number of markers, we created maps based on 41 mutants and a total of 400 deleted elements, allowing different numbers of genotyped markers (30, 50, 71, 90, or 110). (4) Finally, to evaluate the influence of PCR errors, we created maps based on 71 markers, 41 mutants, and 400 deleted elements, but with different numbers of deleted elements (10, 30, or 50) added randomly to the simulated ones, which did not affect lengths of original deletions. Thirty maps were created and used as input for DelMapper for each of the situations listed above. We recorded the percentages of trials that successfully reconstructed the input marker order as well as the number of estimated maps with the best score.

### Software availability

DelMapper and DelMapmaker are available at https://github.com/ion-beam-breeding/DelMapper and https://github.com/ion-beam-breeding/DelMapMaker, respectively.

## Additional Information

**How to cite this article**: Kazama, Y. *et al.* A new physical mapping approach refines the sex-determining gene positions on the *Silene latifolia* Y-chromosome. *Sci. Rep.* **6**, 18917; doi: 10.1038/srep18917 (2016).

## Supplementary Material

Supplementary Information
