# Supplementary material for: A new physical mapping approach refines the sex-determining gene positions on the Silene latifolia Y-chromosome
Source: Sci Rep. 2016 Jan 8;6:18917. doi: 10.1038/srep18917 (PMC4705512; doi:10.1038/srep18917)
Supplement: Supplementary Information [file srep18917-s1.pdf]

Supplementary Information: A new physical mapping approach refines the sex-determining gene positions on the *Silene latifolia* Y-chromosome

Authors: Yusuke Kazama[1,*], Kotaro Ishii[1], Wataru Aonuma[2], Tokihiro Ikeda[1], Hiroki Kawamoto[2], Ayako Koizumi[2], Dmitry Filatov[3], Margarita Chibalina[3], Roberta Bergero[4], Deborah Charlesworth[4], Tomoko Abe[1], and Shigeyuki Kawano[2]

Affiliations:

[1] RIKEN Nishina Center, 2-1 Hirosawa, Wako, Saitama 351-0198, Japan

[2]Department of Integrated Sciences, Graduate School of Frontier Sciences, The University of Tokyo, Kashiwa, Chiba 277-8562, Japan

[3]Department of Plant Sciences, University of Oxford, South Parks Road, Oxford, UK

[4]Institute of Evolutionary Biology, University of Edinburgh, School of Biological Sciences, Edinburgh EH9 3JT, UK

[*] Correspondence and requests for materials should be addressed to Y.K. (email: ykaze@riken.jp).

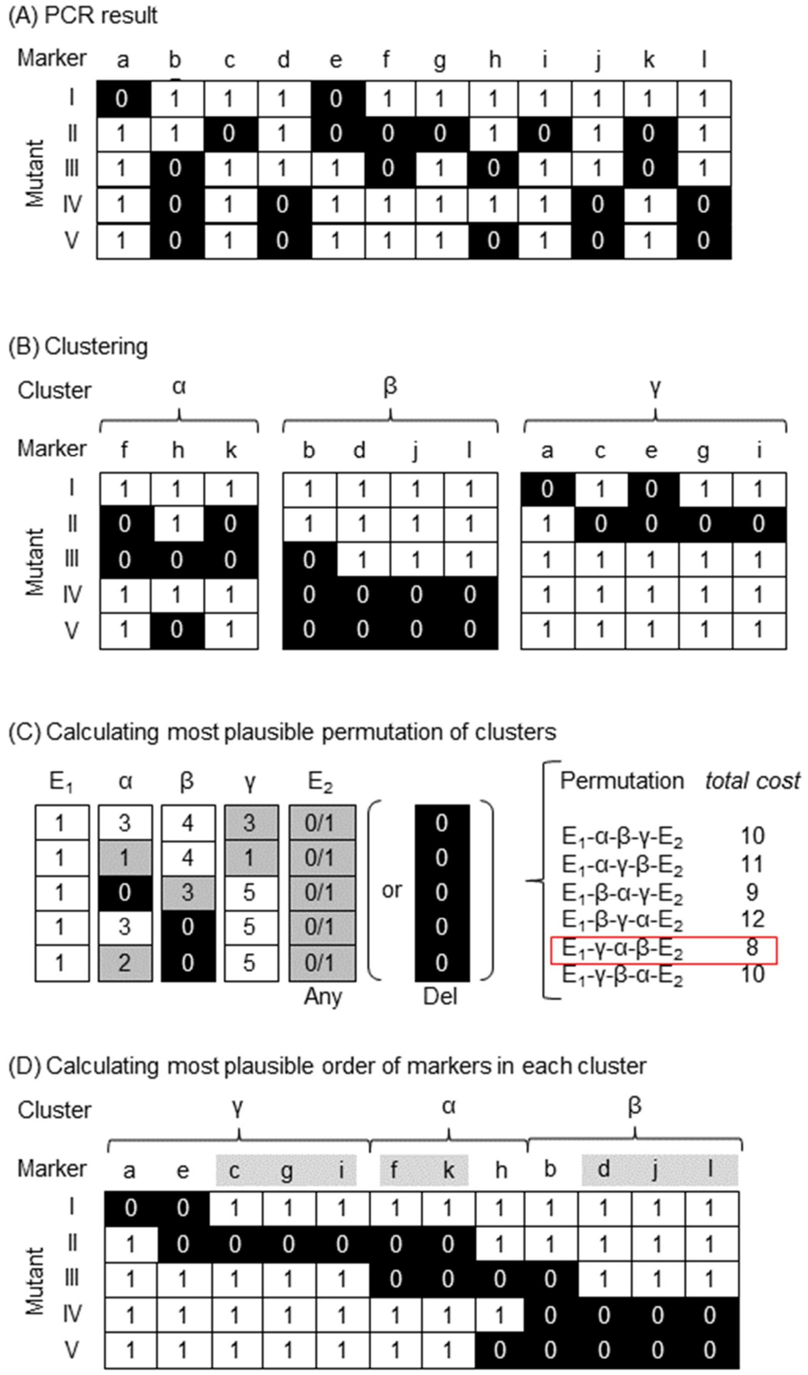

Supplementary Figure 1 | Schematic diagram for deletion mapping procedure using DelMapper. An example of mapping using a data set of 5 mutants and 12 markers is shown. (a) A chart of the data set provided by PCR. The “0” or “1” means that the marker is absent or present in the mutant, respectively. (b) The 12 markers are classified into three clusters to reduce the computational effort. (c) The markers in each cluster are treated as a single virtual marker which can have multiple states (the number of present

markers in the cluster). If the number of markers in a cluster exceeds three and only one marker is absent in the cluster, the absent markers is neglected as a PCR error. The most plausible order of clusters with lowest *total cost* (sum of breakages) is determined from all permutations. The status of both ends (E_1 and E_2) is set according to the Del/Any options (See Methods). (d) After deciding the most plausible order of clusters, the most plausible order of the markers in each cluster is decided in the same manner as (c). For the markers highlighted in grey, the orders do not affect the value of *total cost*.

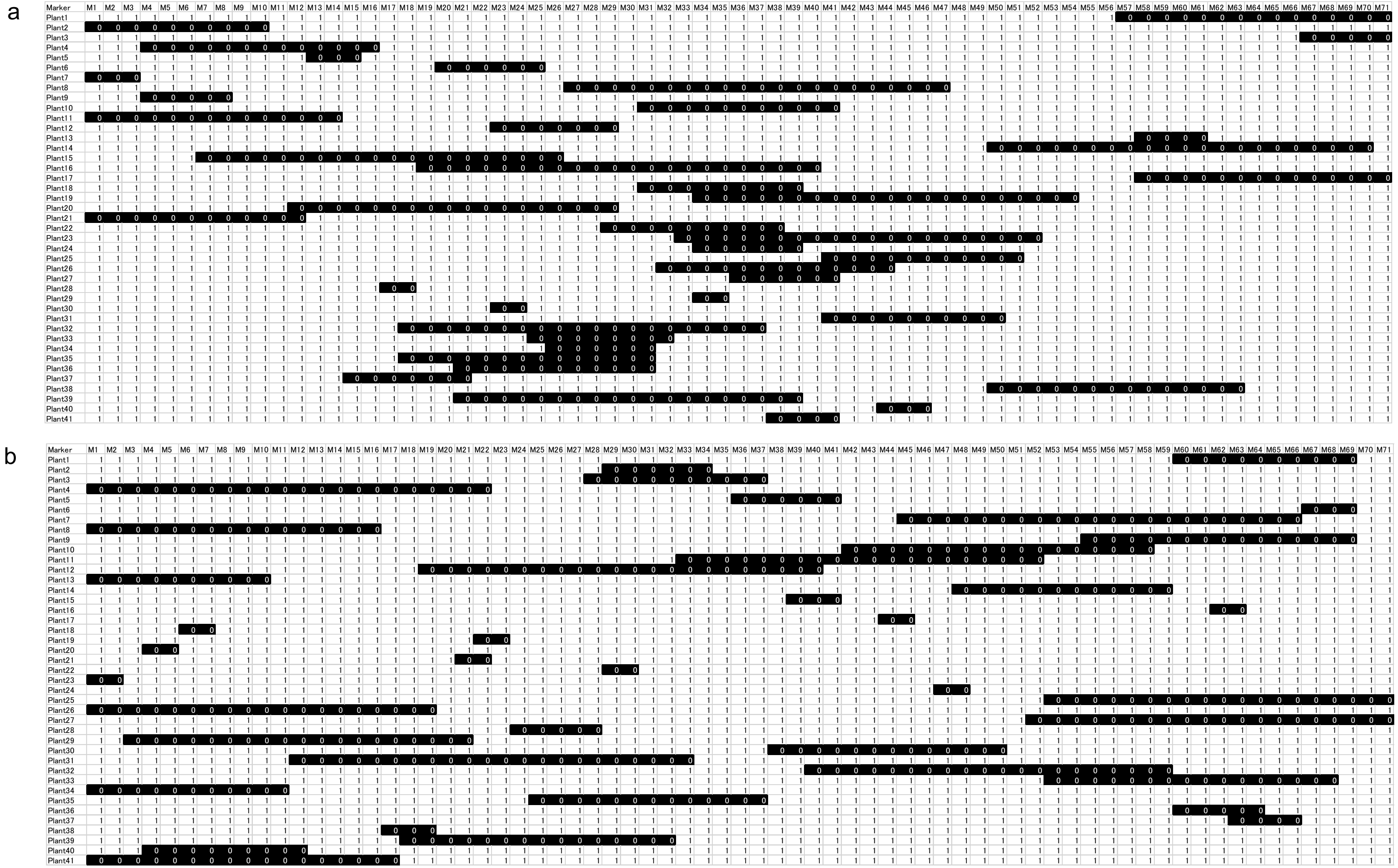

Supplementary Figure 2 | Examples of computer-generated maps. (a) An example of a map with bias in the positions of deletions. When these data were used as input for our program and the analysis was run with the “Del” option, the correct map was not obtained due to the concentration of deletions to the left end of the chromosome. (b) An example of a map without bias in the deletion positions.

	GSF	MK17	L10	Slss	Bgl10	L9	DD44	L12	L13	L16	L14	L11	L15	MS4	ScD05	SlAP3Y	SlY3	C1	L26	SPF	ScQ14	C1	SlY4	MFF	L3_L4	L5	L6	L7	L8	SlY1
cluster	1	1	2	3	4	4	4	5	6	6	7	8	8	9	10	11	12													
UH8	0	0	0	0	0	0	0	0	0	0	0	0	0	0	0	0	1	1	1	1	1	1	1	1	1	1	0	0	0	0
UH15	0	0	0	0	0	0	0	0	0	0	0	0	0	0	0	0	0	1	1	1	1	1	1	1	1	1	0	0	0	0
UH4	0	0	0	0	0	0	0	0	0	1	0	0	0	1	1	1	1	1	1	1	1	1	1	1	1	1	0	0	0	0
MH78	0	0	0	0	0	0	0	0	0	0	0	0	0	0	0	1	1	1	1	1	1	1	1	1	1	1	1	1	1	1
MH14	0	0	0	0	0	0	0	0	0	0	0	1	1	1	1	1	1	1	1	1	1	1	1	1	1	1	1	1	1	1
UH7	0	0	0	0	0	0	0	1	1	1	1	1	1	1	1	1	1	1	1	1	1	1	1	1	1	1	1	1	1	1
MH79	0	0	0	0	0	1	1	1	1	1	1	1	1	1	1	1	1	1	1	1	1	1	1	1	1	1	1	1	1	1
MH115	0	0	0	0	1	1	1	1	1	1	1	1	1	1	0	0	0	1	1	1	1	1	1	1	1	1	1	1	1	1
MH12	0	0	1	0	1	1	1	1	1	1	1	1	1	1	1	1	1	1	1	1	1	1	1	1	0	0	0	0	1	1
UH17	0	0	0	1	1	1	1	1	0	0	0	1	1	1	1	1	1	1	1	1	1	1	1	1	1	1	0	1	1	1
UH9	0	0	0	1	1	1	1	1	1	1	1	1	1	1	1	1	0	1	1	1	1	1	1	1	1	1	1	1	1	1
MH5	0	0	0	1	1	1	1	1	0	0	0	1	1	1	1	1	0	1	1	1	1	1	1	1	1	1	1	1	1	1
UH13	0	1	1	1	1	1	1	1	1	0	0	1	1	1	1	1	1	1	1	1	1	1	1	1	1	1	1	1	1	1
MS36	1	1	1	1	1	1	1	1	1	1	1	1	1	1	1	1	1	1	0	0	0	1	1	1	1	1	1	1	1	1
MS37	1	1	1	1	1	1	1	1	1	1	1	1	1	1	1	1	1	1	0	0	0	1	1	1	1	1	1	1	1	1
MS34	1	1	1	1	1	1	1	1	1	1	1	1	1	1	1	1	1	1	0	0	0	1	1	1	1	1	1	1	1	1
MS75	1	1	1	1	1	1	1	1	1	1	1	1	1	1	0	1	1	1	0	0	0	1	1	1	0	1	1	1	1	1
MS65	1	1	1	1	1	1	1	1	1	1	1	1	1	1	1	1	1	1	1	0	0	1	1	1	1	1	1	1	1	1
MS62	1	1	1	1	1	1	1	1	1	1	1	1	1	1	1	1	1	1	1	1	1	1	0	0	0	0	0	0	1	1
MS63	1	1	1	1	1	1	1	1	1	1	1	1	1	1	1	1	1	1	1	1	1	1	0	0	0	0	0	0	0	0
US9	1	1	1	1	1	1	1	1	1	1	1	1	1	1	1	1	1	1	1	1	1	1	1	0	0	0	1	1	1	1
MS96	1	1	0	1	1	1	1	1	0	0	0	0	0	1	1	1	1	1	1	1	1	1	0	0	1	1	1	1	1	1

Supplementary Figure 3 | Reconstruction of deletion map for *S. latifolia* Y chromosome from previously published deletion markers. The data set provided by Zluvova et al.[31] was input into DelMapper with the "Del" option, resulting in reconstruction of the map identical to previously published one. The closely linked marker sets are coloured grey.

a (k = 1)

Classification of $marker_k$	*State of $marker_{k+1}$	Classification of $marker_{k+1}$	$breaks_m$
present	num_{k+1}	present	0
present	$< num_{k+1}$	deleted	1

b (k > 1)

Classification of $marker_k$	*State of $marker_{k+1}$	Classification of $marker_{k+1}$	$breaks_m$
present	num_{k+1}	present	0
present	$< num_{k+1}$	deleted	1
deleted	> 0	present	1
deleted	0	deleted	0

Supplementary Figure 4 | Diagram showing the method used to determine the quantity of "*breaks*" under the two alternative states of two neighbouring virtual markers. (a) When $k = 1$. Here, the kth marker, representing the end of the mapping region, was classified as deleted. (b) When $k > 1$. *The number of the actual markers in *markerk+1*.

Supplementary Table 1 | List of mutants used in this study.

Mutant ID	Phenotype	Mutagen (dose)	Irradiated tissue	Referrence
R025	Hermaphrodite	C ion (100 Gy)	Dry seed	Koizumi et al.[1]
EGP4	Hermaphrodite	C ion (120 Gy)	Dry seed	This study
EGP5	Hermaphrodite	γ-ray (20 Gy)	pollen	This study
EGP6	Hermaphrodite	C ion (20 Gy)	pollen	This study
EGP7	Hermaphrodite	γ-ray (40 Gy)	pollen	This study
EGP8	Hermaphrodite	γ-ray (20 Gy)	pollen	This study
EGP9	Hermaphrodite	C ion (20 Gy)	pollen	This study
EGP10	Hermaphrodite	C ion (10 Gy)	pollen	This study
EGP11	Hermaphrodite	C ion (20 Gy)	pollen	This study
EGP12	Hermaphrodite	C ion (20 Gy)	pollen	This study
EGP13	Hermaphrodite	C ion (20 Gy)	pollen	This study
EGP14	Hermaphrodite	C ion (20 Gy)	pollen	This study
EGP15	Hermaphrodite	C ion (20 Gy)	pollen	This study
EGP19	Hermaphrodite	C ion (20 Gy)	pollen	This study
mk17-2	Hermaphrodite	γ-ray (20 Gy)	pollen	This study
GPSS1	Female like	γ-ray (20 Gy)	pollen	This study
K034	Asexual	Spontanious		Koizumi et al.[2]
ESS1	Asexual	C ion (100 Gy)	Dry seed	This study
ESS2	Asexual	C ion (100 Gy)	Dry seed	This study
ESS3	Asexual	C ion (40 Gy)	pollen	This study
ESS4	Asexual	C ion (20 Gy)	pollen	This study
ESS5	Asexual	C ion (20 Gy)	pollen	This study
ESS6	Asexual	C ion (100 Gy)	pollen	This study
ESS7	Asexual	C ion (20 Gy)	pollen	This study
ESS8	Asexual	C ion (20 Gy)	pollen	This study
scq14-1	Asexual	C ion (20 Gy)	pollen	This study
ISS1	Anther defect	C ion (100 Gy)	Dry seed	This study
ISS3	Anther defect	C ion (100 Gy)	Dry seed	This study
ISS5	Anther defect	C ion (20 Gy)	pollen	This study
ISS6	Anther defect	C ion (20 Gy)	pollen	This study
ISS7	Anther defect	C ion (20 Gy)	pollen	This study
ISS8	Anther defect	C ion (20 Gy)	pollen	This study
ISS9	Anther defect	C ion (20 Gy)	pollen	This study
ISS10	Anther defect	C ion (20 Gy)	pollen	This study
ISS11	Anther defect	C ion (20 Gy)	pollen	This study
LSS1	Pollen defect	γ-ray (40 Gy)	pollen	This study
LSS2	Pollen defect	γ-ray (80 Gy)	pollen	This study
LSS3	Pollen defect	γ-ray (20 Gy)	pollen	This study
LSS4	Pollen defect	γ-ray (20 Gy)	pollen	This study
LSS5	Pollen defect	γ-ray (40 Gy)	pollen	This study
slap3y-3		C ion (20 Gy)	pollen	This study

Supplementary Table 2 | List of STS markers used in this study.

Name	Anealing temperature	Forwad primer sequence from 5' to 3'	Reverse primer sequence from 5' to 3'
BGL10	55	ATCACCTTCCACCTTCACGC	AAATGCGGCCAGGCTAACAG
BGL16	56	TCATGGGGTTAGCAGAAAGG	TGCTTAGGAAATTGCGATCC
contig01089Y	60	GACAAGAGATTGGGAACCTTCAGG	CGGGTGTAGATCGGTTACTATTGG
contig01402Y	60	GTTGGTTACAAGAATCTTCCTTGCCC	CTGTGCCCTATAATCAGTTCCCTT
contig01455Y	60	CCCATCTCTACATAAGAGTAAGACGG	CCCCTTCAGGAAGTTTAGCAACC
contig02064Y	60	CTATCGGCCAGGCACAC	TGTTTGAATGTATGTAGCAGCCC
contig02096Y	60	CAAGGCTTCAGCACCAAAAGA	AAGCTCAGAAGGAGGAGTG
contig02118Y	60	ACAGTTTTGCCACCCCACT	AGCTTCATTAAGAACCCCCC
contig02417Y	60	CCCCTTGTGATTAGTTTAGTTATAGG	AGGAGGTGTTGGGGGAG
contig03376Y	60	ACTTGATATCAAAAATCTCCGTGAC	CGACTGTGGAGGTATGTTG
contig03837Y	60	GGTATCAAGGGTTTAACCAAGTT	GGGTTTTATACCTGCTTCAAGAA
contig03968Y	60	GGCGACAAGCTCGAGCT	GGGAATGAACAACACCGTC
contig04032Y	60	ATTAGAGAAGGAGCGCGAG	GCTATCTTCAAGCTGCATTTTCAA
contig04554Y	60	GCGGCTAAATTCTATTAGAAGAG	GTGTTCAACACCCCCGAC
contig04719Y	60	CCCTACTAAAGTCAACAACTTGATA	GCCTCTTTCAATTCTGTGCC
contig05982Y	60	AGGTGGCCTGAGAAGGAA	GCTCTGCTTACTCTCCAG
contig06011Y	60	CGCGGATCAATGCTTGGAATA	CGGCGCCAAGACCTTGA
contig06349Y	60	ATTCTGGTAAGGATGCTGGG	GTGGCAACAACAGCCATTTC
contig06721Y	65	CGTGGCTAGCTATTGTTAACT	GCGTCCCCTTACAGCCTT
contig06843Y	60	CTGACATTGCCACTAATACTATG	ATGTAGATCTAATGTTTTGGTGTC
contig08194Y	60	GCACAAGGTGAATTTCGATGC	TTTGCAGAGGACGCATCAGTTTTA
contig08525Y	60	CCTATTTTGAAACGCGTTACTG	TAGGCTGTTACCATTGACCA
contig08635Y	60	CAAGCTCTCCGTTGTGG	ATACGTCTCATTTTTCATGATGC
contig08645Y	60	TTGAGCTTGTCCTCTGGGT	CGAGTCCTCAAATCATCCTCA
contig09144Y	60	CAGCGTTTGGACTTTACTGTAG	CCCCAGGGTTTGAATCTCA
contig09315Y	60	CTACACTTTACAGGCAGAAAGC	CAGACTTAAAGTAAGAACGTTGTTG
contig12985Y	60	CATCCGACCAAAATGAAAATGCC	CGAGTCAGGTAGAGTTGCT
contig14965Y	65	CAACCGCTCACGTCTCC	CAAATCCTTATCTAAATGGGCCA
contig15251Y	60	CCAACCTTCACCACAACCAT	TAAGGAAGAAGATGAAGAGAGAC
contig16145Y	60	TCTTAAATTGGTGAGGAAGCATG	CCATGCACCAAATCATTCTGG
contig17233Y	60	GATAGGCAGAGAAGAACTGC	AGGGTCAAGATCGCGTTG
contig17931Y	60	TTGAATCATGGACGACCGC	TTTGTTCTTTGGAATTGATGCCC
contig19298Y	60	CGACCAAGATAAACTTGTTGGTC	TGAGGAGGTCATTCCCACTA
contig19402Y	60	GAAACTGAGGACATTGATACAAAAG	GTCGATGTTACACCTGCACA
contig20685Y	60	GACTTGCTGCTAAGTCAAGG	ATGCGTCATCGCTTAATTCAAATGCT
contig22556Y	60	GGGTGGTATCCCTACAAAC	TGAATTTCTTAATTTGTCCAGCCAC
contig23003Y	60	GTTGGATCATTCTGGTCTTTGC	CTGGAACCCCTCTAACCA
contig23089Y	60	GATAAAGTGAAGTTGGCACGG	TGAAAATATGGCATAATGGTAAAGTGTT
contig23128Y	60	CGTCTCGGCTTTGGTTTAC	GAGATAAAGGCTGCTGAACG
contig23217Y	60	CCTTCTTCGTATGATGATGGTG	ACATCAAAGCGTTCACTAGGAG
contig23440Y	60	CCAGAATTTGCAGTTACACAGG	CCACTTCTTTTCGCCGTGTTTT
contig23791Y	60	GCTTTGGCTCTCAGAGATTTAGA	CAAACGCATCAACTCGATGG
contig24006Y	60	TAGCTTTGGGGGCTCAAATATAG	TCCAGCTTGTTCGCAGAG
contig24682Y	60	CGGCAAATAGAGCAATTAGTGG	CAATACGAAATAACTCGATAAGATCA

Supplementary Table 2 continued.

Name	Anealing temperature	Forwad primer sequence from 5' to 3'	Reverse primer sequence from 5' to 3'
DD44Y	60	CACAGGCGGAGTTACCTCAT	CCCAATGGCTCACTCTTGAT
MK17	56	GGCAGATGTGGGTAATTGCT	GGACTAGAAGGTAACACGGGAAG
MS2	58	ATGACGGACCCTACATTTGG	AGGCGTTGACAAGGAGTATT
MS4	58	AGGCGTTGACCAGTTCAG	GGACACGATGACACCAAC
MS7	58	GATGACGGACCATATGAG	CGCTGACTTCCCCTTACA
ScD05	66	TGAGCGGACACGGGTGGGGC	TGAGCGGACATTGTGAGGTTACCTCC
ScD12	60	TTCCCTCCTCCTTTCTCTCTC	TAGAAAGAAGATGGGTGATTTGG
ScK02	60	GCAAATGGGTTTAGTGTAGTGGTT	GTCTCCGCAATTATCACACTAAGT
ScQ14	67	GGACGCTTCATGACCCATTTACTC	GGACGCTTCAGCGGGCGGGATT
ScX11	58	GGAGCCTCAGGGATTAGAAAGCCT	GGAGCCTCAGTACTAATAACATCA
SlAP3Y	60	GGCATGGAGATCTCCTCATGGATC	TATATTCGAGACAACATGGCCTGG
SlCypY	65	CATGTTGTCGTCTCCTGTGC	GAGATGGGATCTAAAATGCTTTGC
SlssY	58	GTCCGTTGCAAAGGCTCTTC	ACTCACGGACAGGTCTTTTGC
SlY1	66	ATGTAGATTCTGGAAGATCCCCTTG	GGCCAGGCTCATTTTCAAGTAAATG
SlY3	60	CCTTACTGCTGCTGCTCAGGATTATC	ATGCCCTGAGCAGCAGTAAGGCGAAC
SlY4	65	CAACCTGACTTCTCCGCTCCTTCTGG	CAACATGAGCTCCTCGTGAGCACGGCG
SlY6a	60	CACTCTAAGGTTATGAGATTCTAGTTCTG	GGACATGTTGAAAACCTAACCAACGAG
SlY9	55	CTTGTGGAACTTCTGGTGGAAG	GTCCAATCACATTCAAGTCTCTCC
SmicSy1	58	CTCACCGTAGCCGAGAAGAAGGAGAAAGG	AACAACAACAACAACAACAATAATAAT
SmicSy2	55	TGTCGATCGTTCAAAGCAACTACAGG	AACAACAACAACAACAACAATAATAAT
SmicSy3	60	GCTCCCAACACTACGCCTTA	AACAACAACAACAACAACAATAATAAT
SmicSy4	60	GCAAATGAAATCATCTCGACTG	AACAACAACAACAACAACAAGAAGAAG
SmicSy5	60	AGTCGAGAGGCACGAAAATG	AACAACAACAACAACAACAAGAAGAAG
SmicSy6	55	CCATTTCAATTTGGGGTTTG	AACAACAACAACAACAACAAGAAGAAG
Y202(SlY1)	58	GACCTTCCGAACGGTTGAAA	GGCATACACAAACAAGCTAC

Supplementary Methods

Mutants used in this study

The mutants were found by observing floral phenotypes in the M_1 or M_2 generations after irradiation treatments, except for three mutants, mk17-2, scq14-1, and slap3-3, which were obtained by PCR-based screening in the M_1 generation using three STS primers for the sequences MK17, ScQ14, and *SlAP3Y*. MK17 and ScQ14 were thought from previous mapping to be physically close to the GSF and SPF genes, respectively (see below). *SlAP3Y* is homologous to the tomato TM6 gene, which is involved in anther development[3]. The tissues irradiated and the doses used to generate each mutant are listed in Supplementary Table 1. Flowers of wild-type and mutants were observed by scanning electron microscopy (SEM), as described previously[2]. Genomic DNA was extracted from the leaves of male, female, and mutant plants using the DNeasy Plant Mini Kit (Qiagen GmbH, Hilden, Germany).

PCR amplifications of markers for the mapping

A total of 22 previously reported markers showing male-specific amplification in the K line were used in this study[4]. Nine additional markers were tested for male specificity in the K line, of which five markers proved to be useful in these plants: *BGL16*[5], *ScD12*[6], *SlCypY* and *SlY6a*[7], and *SlY9*[8].

In addition, we designed new markers from 73 genes reported by Bergero and Charlesworth[9], whose male specificity was checked. A total of 69 markers were selected at random from these markers (see Supplementary Table 2), and PCR amplifications were performed using Blend Taq polymerase (Toyobo, Tokyo, Japan). The conditions were 5 min at 94°C, followed by 30 cycles of 30 s at 94°C, 30 s at the appropriate temperature for each primer set, and 1 min at 72°C, with a final extension of 5 min at 72°C. Supplementary Table 2 in the supporting information lists the primers used, the annealing temperatures, and references.

FISH analysis of the *SlAp3Y* gene

cDNA of male plants was prepared as previously described[10]. *SlAP3X/Y* cDNA was amplified using the primers 5'-GWK GAA AAA AAT GGG AAG AGG AA-3' and 5'-GCT GCT CYT TCA TTT CCA TAC ATA C-3'. Primers were designed for two neighbouring regions of *SlAP3X* that do not contain repetitive sequences (5'-AAA ACT CGA TCG GTT CAT CTC ATC TCG G-3' and 5'-TGG AAA AGC AAT TCT TAT ACT CGC ACC A-3'; 5'-AGC AAG ACG TAG TAG CAA CGG GTG A-3' and 5'-AGT GGT CTG AAC CGA TTT TGG GTG C-3'), and these were used to amplify these regions from a BAC clone positive for the *SlAP3X* sequence, 13d11E[11]. This process yielded sequences of a region that is non-repetitive in the X-

linked BAC and that should be suitable for FISH analysis to detect *SlAP3X* or *Y* alleles. PCR amplifications were performed in the same manner as those of markers for the mapping. The annealing temperatures were 59°C and 61°C for *SlAP3X/Y* and the two neighbouring regions of *SlAP3X*, respectively. Fluorescent *in situ* hybridisation (FISH) was performed as described previously[12], with minor modifications. Briefly, a probe was prepared from the mixture of the three PCR products using the DIG-Nick Translation Mix (Roche Diagnostics, Basel, Switzerland). Chromosomal DNA was denatured at 70°C for 1 min in 2× SSC buffer containing 70% formamide. The chromosome preparations were dehydrated immediately by 5-min treatments with 70% ethanol at –20°C and 100% ethanol at room temperature. The preparations were then dried for 30 min at room temperature. Each slide was loaded with 10 μL of the hybridisation mixture containing 12.5 ng of probed DNA, 50% formamide, 10% dextran sulphate, and 2× SSC buffer. The slides were washed twice in 50% formamide 2× SSC buffer at 42°. The signals were fluoresceinated with anti-digoxigenin-rhodamine (Roche Diagnostics) for the digoxigenin-labelled probe Preparations were counterstained with 4′,6-diamidino-2-phenylindole (DAPI). The DAPI and rhodamine images were detected consecutively under a Leica Q550 cytogenetic workstation (Leica Microsystems, Wetzlar, Germany) equipped with a black-and-white charged-coupled device camera (CoolSNAP HQ; Nippon Roper, Tokyo, Japan), using the SpectraVision filters SpectrumDAPI and Spectrum-Orange, respectively (Vysis, Chicago, IL, USA).

Supplementary References

1. Koizumi A, *et al.* Two Separate Pathways Including SlCLV1, SlSTM and SlCUC That Control Carpel Development in a Bisexual Mutant of Silene latifolia. *Plant and Cell Physiology* **51**, 282-293 (2010).

2. Koizumi A*, et al.* Floral development of an asexual and female-like mutant carrying two deletions in gynoecium-suppressing and stamen-promoting functional regions on the Y chromosome of the dioecious plant Silene latifolia. *Plant and Cell Physiology* **48**, 1450-1461 (2007).

3. Matsunaga S*, et al.* Duplicative transfer of a MADS box gene to a plant Y chromosome. *Mol Biol Evol* **20**, 1062-1069 (2003).

4. Fujita N*, et al.* Narrowing Down the Mapping of Plant Sex-Determination Regions Using New Y-Chromosome-Specific Markers and Heavy-Ion Beam Irradiation-Induced Y-Deletion Mutants in Silene latifolia. *G3-Genes Genom Genet* **2**, 271-278 (2012).

5. Donnison IS, Siroky J, Vyskot B, Saedler H, Grant SR. Isolation of Y chromosome-specific sequences from Silene latifolia and mapping of male sex-determining genes using representational difference analysis. *Genetics* **144**, 1893-1901 (1996).

6. Zhang YH, *et al.* Y chromosome specific markers and the evolution of dioecy in the genus Silene. *Genome / National Research Council Canada = Genome / Conseil national de recherches Canada* **41**, 141-147 (1998).

7. Bergero R, Forrest A, Kamau E, Charlesworth D. Evolutionary strata on the X chromosomes of the dioecious plant Silene latifolia: Evidence from new sex-linked genes. *Genetics* **175**, 1945-1954 (2007).

8. Kaiser VB, Bergero R, Charlesworth D. A new plant sex-linked gene with high sequence diversity and possible introgression of the X copy. *Heredity* **106**, 339-347 (2011).

9. Bergero R, Charlesworth D. Preservation of the Y Transcriptome in a 10-Million-Year-Old Plant Sex Chromosome System. *Curr Biol* **21**, 1470-1474 (2011).

10. Ishii K, *et al.* The Y chromosome-specific STS marker MS2 and its peripheral regions on the Y chromosome of the dioecious plant Silene latifolia. *Genome / National Research Council Canada = Genome / Conseil national de recherches Canada* **51**, 251-260 (2008).

11. Ishii K, *et al.* Rapid degeneration of noncoding DNA regions surrounding SlAP3X/Y after recombination suppression in the dioecious plant Silene latifolia. *G3* **3**, 2121-2130 (2013).

12. Ishii K, *et al.* Analysis of BAC clones containing homologous sequences on the end of the Xq arm and on chromosome 7 in the dioecious plant Silene latifolia. *Genome / National Research Council Canada = Genome / Conseil national de recherches Canada* **53**, 311-320 (2010).
